# Recyclable and Biobased Vitrimers for Carbon Fibre-Reinforced Composites—A Review

**DOI:** 10.3390/polym16081025

**Published:** 2024-04-09

**Authors:** Hoang T. T. Tran, Shammi Sultana Nisha, Racim Radjef, Mostafa Nikzad, Robert Bjekovic, Bronwyn Fox

**Affiliations:** 1 Department of Mechanical Engineering and Product Design Engineering, Swinburne University of Technology, Melbourne, Victoria 3122, Australia; snisha@swin.edu.au (S.S.N.); rradjef@swin.edu.au (R.R.); mnikzad@swin.edu.au (M.N.); 2 Faculty of Mechanical Engineering, University of Applied Sciences Ravensburg-Weingarten, 88250 Weingarten, Germany; robert.bjekovic@rwu.de

**Keywords:** biopolymer, decarbonization, circular economy, covalent adaptable network, reprocessability, sustainability

## Abstract

Economic and environmental concerns over the accumulation of end-of-life carbon fibre composite waste have led to increased attention to sustainable materials with low environmental impact. Over decades of research, vitrimers, a modern class of covalent adaptable networks, have bridged the gap between thermoplastics and thermosets. With the distinguishing feature of dynamic covalent bonds, vitrimers can be rearranged and reprocessed within their existing network structures in response to external stimuli such as heat or light. This poses a unique solution to repairing damaged composites, extending their service life, and reducing post-consumer waste. However, the synthesis of vitrimers often requires petrochemical consumption, which increases their carbon footprint. Using bio-based materials could be a promising solution to reduce the reliance on petrochemicals and their related pollution. This review compiles the contemporary requirements for bio-based vitrimers regarding their properties, scalability, and recycling features. This article also presents a comprehensive overview of the pathways to produce sustainable bio-based vitrimers and an overview of promising studies showing the potential uses of bio-derived vitrimers on carbon fibre composite productions.

## 1. Introduction

Carbon fibre-reinforced composites (CFRC) are advanced non-metallic composite materials constituting polymer resin and carbon fibres, in which the carbon fibres act as the reinforced materials and the polymer resin acts as the matrix holding the fibres [1,2]. Thanks to their high stiffness, lightweight, and excellent fatigue resistance, CFRCs have been increasingly utilised in aerospace, automotive, marine, sports, and renewable energy industries [3,4,5]. The global market is expected to go up to USD 126.3 billion by 2026 [6]. Moreover, CFRC is considered a potential alternative to replace metal parts (e.g., steel, iron, aluminium, titanium, alloys, etc.) in building construction, aircraft, automotive, and wind energy applications, with outstanding high strength, stiffness, and lightweight properties, which results in lower greenhouse gas emission and closer toward the path of decarbonisation strategy [2,7]. For instance, a total of 50% of the Boeing 787 and Airbus A350 aeroplane bodies are made up of CFRC [8].

However, such a soaring demand for CFRCs leads to a significant increase in its related waste, deriving from both manufacturing processes and end-of-life materials. Indeed, the CFRC waste from the manufacturing process (prepregs scraps, cut-offs, etc.) can take up to 40 wt.% of the total materials, and CFRC end-of-life waste is predicted to reach up to 200,000 tonnes annually by 2025 [7,9]. For instance, about 12,000 aircraft will reach their end of service life within the next two decades, generating a massive amount of composite waste [7]. Moreover, a major drawback of CFRCs is their lack of recyclability. Specifically, thermosetting resins—one of the most widely used resin matrices for CFRC productions due to their high mechanical durability, thermal stability, and corrosion resistance—are inherently difficult to reprocess and recycle. This is because of their permanent three-dimensional cross-linked structures, ultimately resulting in great economic and environmental concerns [10,11]. Landfill disposal and incineration are two common traditional treatments for CFRC waste due to their simple and low-cost procedures. However, these methods pose high risks of environmental pollution and have been legally banned by many countries [3]. Other recycling technologies (e.g., grinding, solvolysis, pyrolysis, fluidized bed, supercritical hydrolysis, etc.) have widely been applied in the last two decades. Nevertheless, these approaches have high energy and operational cost requirements, and the recovered carbon fibres are reduced in size and exhibit deteriorated mechanical properties [3,9]. Therefore, the exploration of non-destructive and closed-loop recovery technologies for CFRC productions is vital for the “zero waste” approach and circular economy model in CFRC industries.

**Figure 1:**
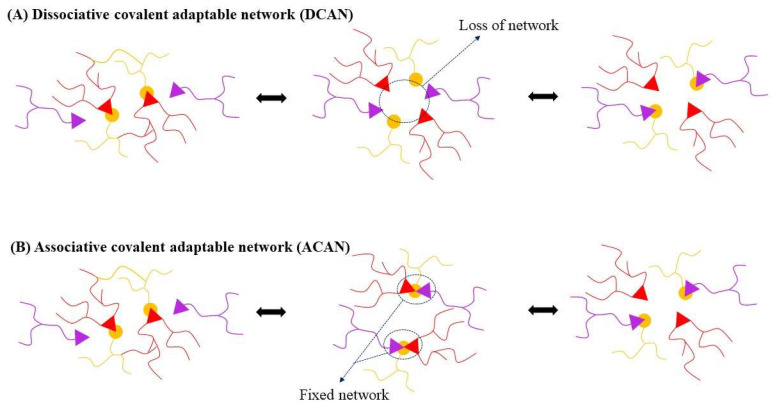
(**A**) Dissociative exchange covalent adaptable networks CANs, and (**B**) Associative exchange CANs include “vitrimers”—contain dynamic bonds that can break and form concurrently, maintaining fixed cross-linked density.

Global attention has been recently directed to reprocessable and recyclable CFRC based on degradable thermosetting resins (i.e., recycled plastics). The EU Commission has proposed a new regulation on the design, production and end-of-life treatment of vehicles in terms of reusability, recyclability and recoverability, which is expected to reduce 12.3 million tons of carbon dioxide (CO_2_) emission by 2035, valorise 5.4 million tons of materials, as well as increase the recovery of critical materials. Moreover, 25% of plastic used to build a new vehicle will be required to come from recycling, of which 25% should be recovered from post-consumer vehicle wastes. Therefore, recent years have witnessed the development of recyclable thermosets incorporated with covalent adaptable networks (CANs). These polymers contain dynamic covalent bonds that can rearrange the polymer network structures in response to external stimuli (e.g., temperature, pH, solvents, light, etc.), enabling them to behave as both thermosets at service temperature and thermoplastics at elevated temperature. Based on the exchange mechanism, the covalent adaptable network polymers are categorised into two groups: dissociative CANs and associative CANs. The difference between the dissociative and associative exchange mechanisms is illustrated in Figure 1. The dissociative CANs rely on dynamic bonds that break first, followed by reconnecting with new linkages at different positions in the polymer network, resulting in the loss of viscosity, lack of solvent resistance, and reduced dimensional stability [12]. While the associative CANs—also known as “vitrimer”—contain dynamic bonds that can break and form concurrently, maintaining fixed cross-linked density and exhibiting excellent solvent resistance [12].

**Figure 2:**
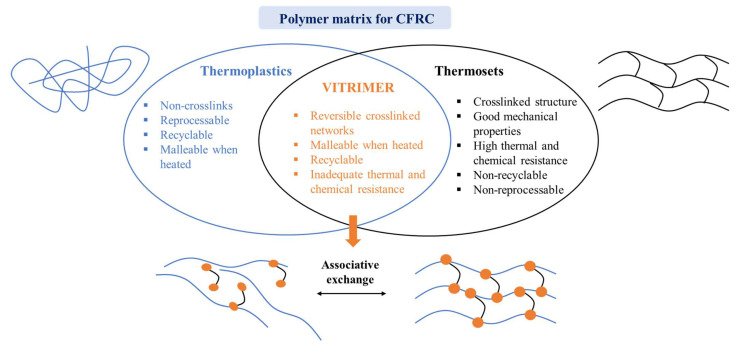
Vitrimer is a third class of polymer matrix for composites, bridging the gap between thermoplastics and thermosets.

Vitrimers are categorised as a third class of polymeric materials, followed by two conventional polymers which are thermoplastics and thermosets (Figure 2) [13]. Possessing outstanding shape memory, self-healing, and reprocessing properties, vitrimers are able to act as both thermosets at service temperature and thermoplastics at elevated temperature [14]. These distinctive features make vitrimers an attractive alternative to polymer resins for CFRC materials. Since the first vitrimer was synthesised through a transesterification exchange reaction [15], several vitrimers have been prepared through a variety of dynamic exchangeable reactions such as transcarbonation of carbonates [16], transesterification of esters [17,18,19], transamination of vinylogous urethanes [20], imine bonds exchange [21], disulfide exchange [22], etc. However, most current vitrimers, especially for high-end CFRC applications, are heavily dependent on petroleum resources, thus leading to economic and environmental consequences. To respond to worldwide concerns over the scarcity of fossil fuel and environmental pollution, the exploration of bio-based vitrimers has become a promising objective for the development of green and sustainable CFRCs. Several bio-derived vitrimers have been investigated through the exploitation of various kinds of renewable feedstocks including cellulose [13], fructose [23], fruit juices [24], vegetable oils [14,25,26,27], and lignin-derived vanillin [3,28], etc. Some biobased vitrimers have demonstrated performance levels in areas such as thermal stability and mechanical properties, which are comparable to the conventional thermosets [29,30]. The development of vitrimers from natural resources is now attracting enormous attention as a closer step towards green and sustainable composite materials. Therefore, it is of great importance to review recent approaches in the synthesis and characterisation of bio-derived vitrimers as well as their applications in CFRCs.

Herein, this review article firstly provides an overview of potential alternative feedstocks (e.g., vegetable oils, carbohydrates, lignocellulosic-derived chemicals, etc.), which are natural and highly abundant for bio-based vitrimer production. Secondly, sustainable approaches using dynamic covalent chemistry to synthesise vitrimers from renewable materials are examined. Thirdly, the applications of bio-based vitrimers in CFRCs are discussed. Finally, research challenges and future perspectives conclude the review.

## 2. Potential Feedstocks for Bio-Based Vitrimer Synthesis

**Figure 3:**
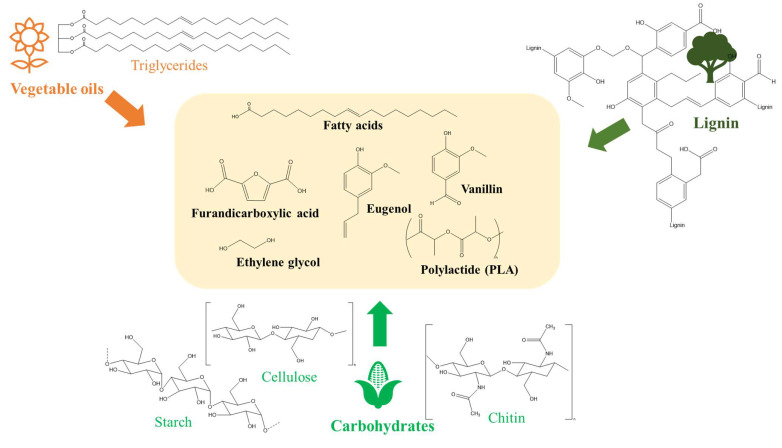
Potential renewable resources for the synthesis of bio-based vitrimers.

With the increasing concerns regarding the rapid depletion of fossil fuels and the environmental problems associated with the use of fossil fuels and petrochemicals, several bio-based vitrimers have been explored lately. A variety of natural resources such as vegetable oils [26,31], lignin or lignin-derived compounds [32,33], fatty acids [34], etc., have been explored for the synthesis of bio-based vitrimers (Figure 3). The incorporation of these green materials would not only be useful to reduce the production cost but also provide a facile approach to extend the utilisation of bio-based vitrimers in different fields.

### 2.1. Vegetable Oils/Epoxidized Vegetable Oils

**Figure 4:**
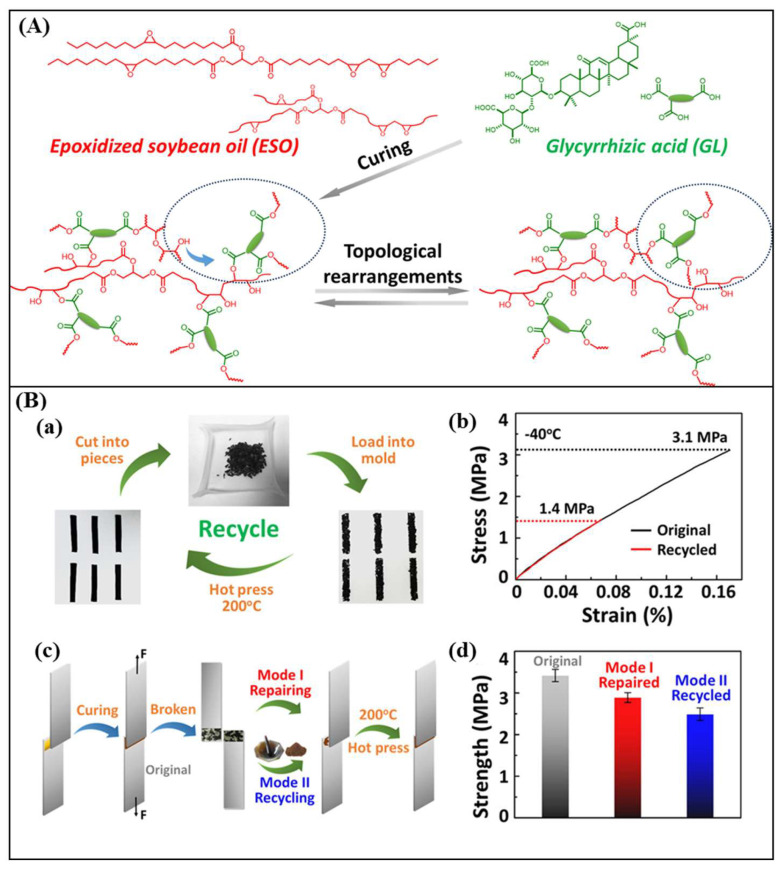
(**A**) Synthesis of ESO–GL–based vitrimer, and (**B**) recycling process of ESO–GL–based vitrimer. (**a**) Photos showing the recycling process of ESO–GL vitrimers, (**b**) stress-strain curves of ESO–GL vitrimers at – 40 °C before and after recycling, (**c**) Schematic view of lap-shear tests for ESO–GL vitrimers, and (**d**) Lap-shear strength of original, repaired, and recycled vitrimers. Adapted with permission from [39].

Vegetable oils are considered viable platform chemicals for biopolymers owing to the high amount of fatty acids and diverse functional groups (double bonds, esters, hydroxyl, etc.), low cost, and wide abundance [35,36]. Unsaturated vegetable oils such as soybean, canola (rapeseed), sunflower, hemp, cottonseed, palm oil, safflower oil, etc., mainly consist of triglycerides, containing the esters of glycerol and three fatty acids [37] (Figure 3). Triglycerides can be directly utilized in the production of polymer resins, with relatively low glass transition temperatures. For example, glycerine polyurethane and triethylene tetraamine polyurea showed glass transition temperatures at 19 °C and 31 °C, respectively [38]. In order to enhance the chemical reactivity, vegetable oils are usually chemically treated to produce epoxidized oils, which can be cured into an epoxy resin system, in similar ways as curing with petroleum-derived counterparts [37]. Epoxidized vegetable oils can also be further modified with several chemical reactions (e.g., hydrolysis, hydrogenation, alcoholysis, acrylation, etc.) to open the epoxy ring, producing smaller fatty acids and improving their properties and reactivities [37]. Epoxidized soybean oil (ESO)—a safe, low-cost, and commercially available monomer—has been extensively employed in the synthesis of bio-based vitrimers. For example, Zych and co-workers [36] developed environmentally friendly vitrimers by thiol-acrylate coupling between ESO acrylate and a diboronic ester dithiol. The produced vitrimers showed good reprocessability in multiple cycles without reducing their mechanical strength. They could also perform self-healing of abrasion-related defects at room temperature, owing to their low glass transition temperature (10.5 °C) and rapid boronic ester exchange. Although this ESO-based vitrimer could be a prime example to solve the recycling issues of traditional thermosets, its application as a matrix for high-performance composites is limited due to the low glass transition temperature properties. Thus, several efforts have been made to improve the T_g_ of ESO-based vitrimers by incorporating ESO with acid as a curing agent via transesterification reaction. For instance, Wu et al. [39] developed a fully bio-based and recyclable vitrimer from ESO and a natural glycyrrhizic acid curing agent (Figure 4). The vitrimers exhibited good welding, repairing, and shape memory properties. They also showed excellent degradability by ethylene glycol solvent. The glass transition temperature could be evaluated from 39 to 64 °C depending on the molar ratio of ESO and the acid.

Another ESO-based vitrimer was prepared using fumaropimaric acid as a curing agent and zinc acetylacetonate as a transesterification catalyst [14]. The prepared vitrimer exhibited T_g_ of 65 °C and tensile strength at 16 MPa. In another study, a Schiff base containing vanillin (VA) was used as a dynamic curing agent in the synthesis of ESO-based epoxy vitrimers [25]. The resulting ESO-VA vitrimer was then utilised to produce ESO-VA carbon fibre composites, performing a superior tensile strength (145.4 MPa) and Young’s modulus (1.18 GPa). In addition, ESO-VA composites could be completely recycled under mild acidic conditions without changing their structures and properties. However, the glass transition temperature (T_g_ = 27.6 °C) was still relatively lower than other ESO-based vitrimers [14,39].

It is noted that most reported ESO-based epoxy vitrimers generally exhibit poor tensile strength (4–16 MPa) and low T_g_ (10–65 °C) compared to vitrimers derived from other bio-based materials such as vanillin and eugenol, limiting their utilisation as matrices from composite applications [25,40]. This is because the epoxy groups in ESO are intramolecular compounds, which have great steric hindrance related to the terminal epoxy groups, thereby resulting in low cross-linking density and reducing the performance of the ESO-based vitrimers [41]. Additionally, these ESO epoxy resins are usually involved in costly and complicated monomer preparation and purification [40]. Moreover, most of the epoxy vitrimers prepared from transesterification dynamic chemistry usually require exogenous catalysts to achieve rapid polymer chain exchange. This might diminish the reprocessing and recycling efficiency of the produced vitrimers due to the catalyst instability and leaching under high temperatures [42]. Therefore, the development of bio-based epoxy vitrimers with high performance and cost-effectiveness using vegetable oils as a platform chemical is still a great challenge for the preparation of sustainable and competitive carbon fibre composites.

### 2.2. Carbohydrates

Carbohydrates such as cellulose, hemicellulose, starch, chitin, etc., have been widely studied for the preparation of biopolymers (e.g., packaging, textiles, biomedical nanofibres, etc.) over the past decades due to their natural abundance, low cost, and non-toxicity [43,44,45]. Cellulose, hemicellulose, and starch mainly contain hydroxyl functional groups, which can be further depolymerised into sugar compounds. These compounds are considered precursors of a broad range of important platform monomers such as furan, sorbitol, 5-hydroxymethylfurfural (5-HMF), levulnic acid, lactic acid, etc., for the preparation of bio-based polymers [46]. Additionally, owing to their high strength, stiffness, durability, and biocompatibility, cellulose and hemicellulose have recently emerged as potential alternatives for fabricating new types of bio-based composites [47].

**Figure 5:**
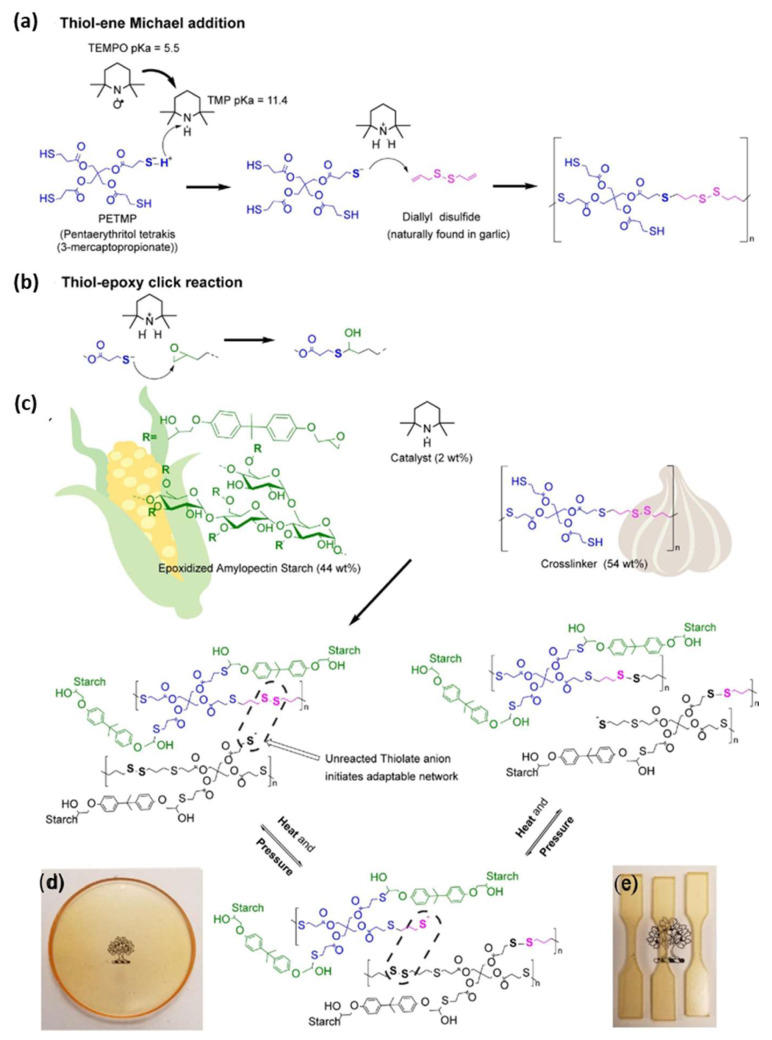
Reaction pathway to prepare a starch-based epoxy vitrimer via exchangeable disulfide bonds. (**a**) Reaction of 3-mercaptopropionate and diallyl disulfide, (**b**) curing mechanism between epoxy and thiol, (**c**) curing mechanism of epoxidized starch and crosslinker, (**d**) cured starch epoxy vitrimer, and (**e**) epoxidized starch epoxy vitrimer cut into dog bone shapes. Adapted with permission from [50].

Considering sustainability and circular economy in the composite industry, a few efforts have been recently made to explore carbohydrate-based vitrimers used as a thermoset matrix in composite materials [48], or as a reinforcement in vitrimer matrix composite [13,49]. For example, Li et al. [48] constructed a new cellulose-based vitrimer by introducing CAN into a carboxymethyl cellulose (CMC) system via dynamic transesterification. This work also incorporated glycerol into the matrix to weaken interchain hydrogen bonds in cellulose, thereby significantly enhancing the mobility of hydroxyls on the CMC and the reprocessability of the produced vitrimers. Zhao and their team reported a novel vitrimer composite using polycarbonate as a matrix and renewable cellulose paper as a reinforcing framework [13]. The hydrogen-bonding interactions between natural cellulose fibres and dynamic polycarbonate resins offered the obtained composite with much improved mechanical strength as well as a series of smart features such as reshaping, self-healing, shape-memory, and reprocessability. In another study reported by Tratnik et al. [50], a starch-based vitrimer epoxy was prepared by exchangeable disulfide bonds and offered potential alternatives to traditional thermosets while improving their lifetime service and providing additional functionalities. In this study, epoxidized amylopectin-rich starch was reacted with a diallyl disulfide and a thiol cross-linker using a click reaction. A detailed reaction for the synthesis of starch-based vitrimer is illustrated in Figure 5. The resulting starch-based vitrimer exhibited outstanding self-healing after reprocessing (990% healing efficiency in tensile strength after five recycling cycles.

Among natural carbohydrates usually applied as fillers for biopolymers, chitin—a primary component of cell walls in fungi and the exoskeleton of shellfish and insects—has been recently explored for the development of green and sustainable layered chitin–vitrimer composites owing to the abundant hydroxyl and N-acetyl groups in its structure [47]. The chitin film was first prepared by an ionic-liquid-assisted method and then embedded into the fatty-acid-derived vitrimer matrix to achieve good interfacial interactions through hydrogen bonding. The obtained chitin–vitrimer composite exhibited good weldability and recyclability under hot-pressing conditions [47].

As one of the most abundant biopolymers on earth, with cellulose and chitin, starch is considered a versatile material for the potential use in biopolymer industries thanks to its hypoallergenicity and hydrogen bonds in semi-crystalline structure, making it easily transformable into thermoplastic materials [51]. Compared to cellulose, starch can be easily converted into smaller chemicals such as ethanol, acetone, organic acids, or other hydroxyl-containing compounds, which can be exploited as monomers or oligomers in the production of biopolymers. However, the application of starch in bio-based polymers remains a challenge because of its instability under various pH, temperature, and shear conditions [52]. Only a few studies have been reported to explore starch-based materials in vitrimer materials. Recently, a sustainable and recyclable starch-derived polymer was developed through transesterification of starch acetoacetate (SAA) with different degrees of substitution (DSs), followed by the solvent-free hot-pressing method [44]. SAA films showed a good range of glass transition temperatures (109–140 °C) depending on the DSs. In addition, SAA films with high DSs (>0.84) exhibited satisfied water insolubility. Also, the maximum tensile strength and elongation at the break of SAA films reached up to 15.5 MPa and 30%, respectively. Nevertheless, water sensitivity, brittleness, and processing difficulty are the main drawbacks of starch, limiting its utilisation in the bio-composite industry [51,52].

### 2.3. Lignin and Derivatives

Lignin is the most abundant resource of renewable aromatic structures in the world and has been widely studied because of its low-cost and highly branched structure, which is considered the most promising renewable natural polymer to replace petroleum-based chemicals. However, it is also the least utilised biomass resource because of its irregular and complex chemical structure and difficulty in isolating the primary lignols as potential precursor to synthesise new recyclable high-performance polymers.

Researchers have emphasized using lignin-based vitrimer (which shows excellent mechanical performance) to increase both the value and utilization of lignin, whilst simultaneously reducing the environmental impact of petroleum products. The vitrimer, which contains an optimized composition of lignin, has very high tensile strength, high elongation at break, very good solvent resistance and multiple recycling options (hot-press recycling, ethylene glycol recycling, alkali recycling) [53]. The lignin-based vitrimer can be prepared via photothermal bonding to avoid the need for organic catalysts or hot-press moulding [53]. Due to its very good solvent resistance, the lignin vitrimer has been used for glass coating or rubber adhesive and can also be easily recycled because of its easy disassembly. The use of lignin for vitrimer production can extend the service life of plastics, reduce the dependence on non-renewable resources, and comply with carbon emission reduction policies, and has a certain potential in green sustainable development strategies.

**Figure 6:**
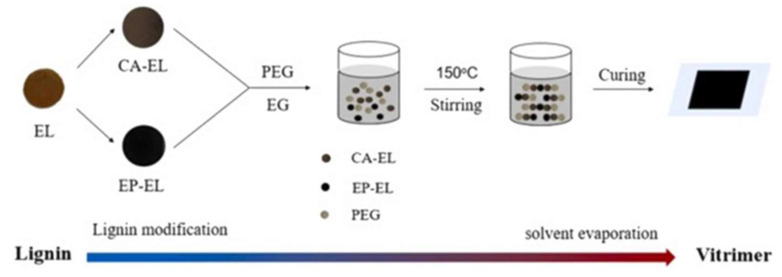
Schematic representation of enzymatic lignin vitrimer synthesis. Adapted with permission from [53].

Du et al. designed a vitrimer that is formed using enzymatic lignin (EL) modified by epoxidation (EP) and carboxylation (CA). The synthesis process of EL-based vitrimer (ELV) is shown below (Figure 6).

Recyclability is a key property of vitrimers. Dynamic covalent bonds are formed within the material, and, under certain conditions, the exchange of dynamic covalent bonds can cause stress relaxation in the material, thus allowing the material to be recycled. Usually, catalysts like TBD or zinc acetate are required to reverse the dynamic ester bond formation, but it is now known that the presence of certain free hydroxyl groups can facilitate ester exchange without the need for catalysts. In this research, the presence of hydroxyl groups in PEG 400 promoted ester exchange, allowing the ELV to undergo stress relaxation without the addition of an esterification catalyst [24] and underpinning the recyclability of the material.

**Figure 7:**
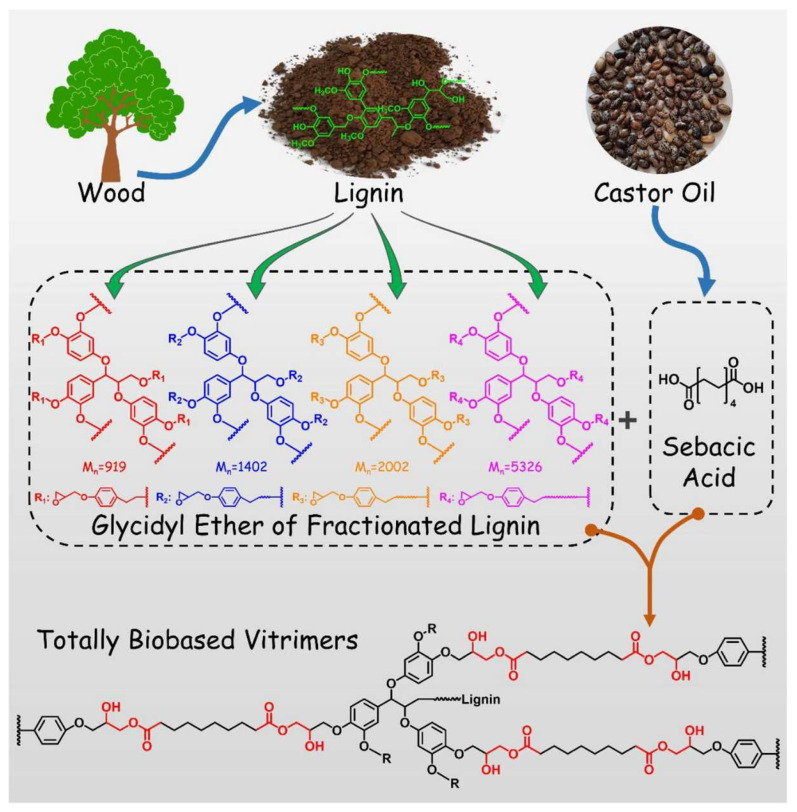
Synthesis of totally biobased vitrimers based on transesterification reactions. Adapted with permission from [54].

Lignin as a natural polymer, shows great potential in preparing functional materials to meet the demand for green/sustainable development. However, the inherent heterogeneity of lignin largely limits its applications. Furthermore, the effect of the lignin complex structure on the performance of the final materials is scarcely mentioned in the literature. Tang and the team [54] successfully prepared, a totally biobased dynamic cross-linked vitrimer with up to 70% lignin content from epoxidized fractionated lignin and sebacic acid without additional chemical modifications (Figure 7).

**Figure 8:**
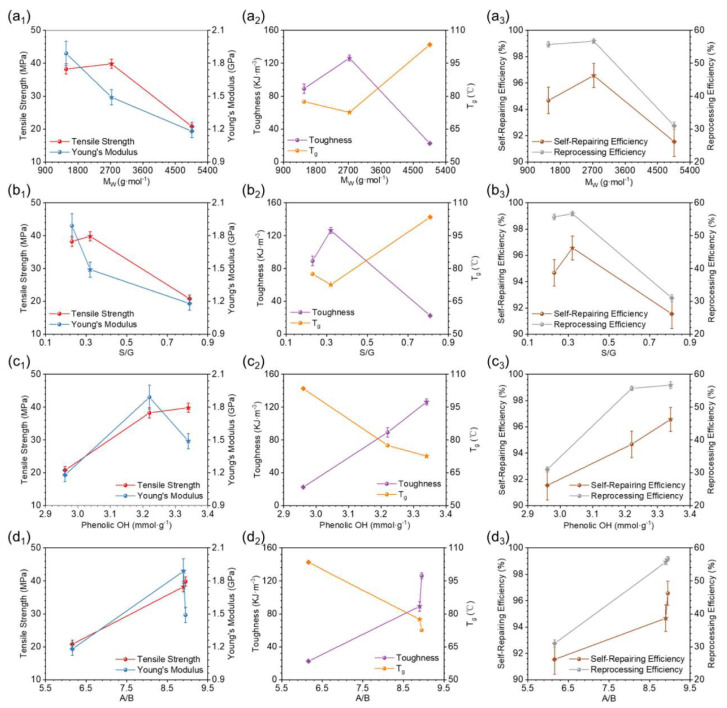
The relationships between the performance of ethyl acetate–soluble lignin LEV_EA_, ethanol-soluble lignin LEV_E_, acetone-soluble lignin LEV_A_ and the lignin molecular weight (**a_1_**–**a_3_**), lignin S/G ratio (**b_1_**–**b_3_**), lignin phenolic hydroxyl content (**c_1_**–**c_3_**), and lignin flexible/rigid linkage ratio (**d_1_**–**d_3_**). In Figure 8, LEV_EA_ is represented by the solid circle, LEV_E_ is represented by the pentagram, and LEV_A_ is represented by the rhombus. A and B mean the amount of β–O–4 and β–5, respectively. Adapted with permission from [54].

The focus of this study was the effect of lignin structure on the performance of the resultant lignin-based epoxy vitrimers (LEVs). The experimental results from the study showed that the properties of resultant LEVs, such as tensile strength, toughness, self-repair ability, and reprocess ability, have a strong correlation with the phenolic hydroxyl content and flexible to rigid linkages ratio in lignin. Meanwhile, strong correlations have also been noticed between the molecular weight of lignin with the thermal properties of the resultant materials. Figure 8 shows the correlation between the performance of LEVs and fractionated lignin structure. As shown in Figure 8a, with increasing Mw of the lignin fractions, the Young’s modulus and tensile strength of LEVs showed a significant decreasing trend, while T_g_ showed the opposite variation tendency. At the same time, the self-repairing and reprocessing efficiencies were less favourable for LEVs with higher molecular weights. The effects of the S/G ratio of fractionated lignin on the properties of LEVs are shown in Figure 8b. Interestingly, the ratio of S/G showed the same effects on the LEV performance as molecular weight, suggesting that the increase in the molecular weights and S/G ratio are detrimental to transesterification.

The phenolic hydroxyl content in lignin has a strong correlation with the thermomechanical properties of the resultant LEVs. As shown in Figure 8c, with the increasing phenolic hydroxyl content of fractionated lignin, the tensile strength and toughness gradually increased. However, their T_g_ showed a decreasing trend (66.8–40.4 °C) due to the high phenolic hydroxyl content in the lignin structure, resulting in more ether bonds and flexible structures in the final structure. Notably, the self-repairing property of LEVs was significantly enhanced with increasing phenolic hydroxyl content in different fractionated lignin. It was found that the crack width on the surface of all LEVs could be reduced by over 90% in 60 s. This finding suggested that high phenolic hydroxyl groups could activate quick recovery of the networks during the reprocessing treatment. The reprocessing properties of different types of LEVs were slightly different. Ethanol-soluble lignin vitrimer (LEV_E_) showed higher reprocessability than the ethyl acetate-soluble lignin vitrimer (LEV_EA_) and acetone-soluble lignin vitrimer (LEV_A_). Furthermore, the flexible/rigid linkage ratio of fractionated lignin also plays a major role in reprocessing property. The increase in molecular weight of fractionated lignin leads to the decrease in reprocessing efficiency to 31.02% due to the low reactivity, high steric hindrance, and the high rigid structure of lignin. As shown in Figure 8d, the higher the ratio of flexible to rigid linkages in the fractionated lignin is, the more favourable the transesterification reaction in the LEVs.

It has also been observed that the LEVs prepared from epoxidized fractionated lignin and sebacic acid exhibited excellent mechanical properties and self-repairing and reprocessing features, with the highest tensile strength of 39.82 MPa and self-repairing efficiency of 96.57%. Moreover, a higher phenolic hydroxyl content and flexible/rigid linkage ratio of fractionated lignin are crucial for obtaining LEVs with excellent self-repairing and reprocessing performances. Such correlations can serve as the basis for the development of green/sustainable and convenient strategies to totally prepare biomass-based LEVs with excellent properties. This work showcases the importance of regulating the heterogeneity of lignin, which is highly related to the properties of the resultant LEVs.

Lignin-based vitrimers are also produced via simple and material-efficient synthetic routes as a biobased circular material for industrial scalability. Here, a series of lignin-based vitrimers built on dynamic acetal covalent networks with a gel content exceeding 95% were successfully prepared in a one-pot, thermally activated, and catalyst-free “click” addition of softwood kraft lignin (SKL) to poly(ethylene glycol) divinyl ether (PDV). Lignin content variation (28 to 50 wt.%) demonstrated the superficial way of tuning the mechanical properties of vitrimers. The lowest lignin content (28 wt.%) showed a tensile strength of 3.3 MPa with a 35% elongation at break, while the corresponding values were 50.9 MPa and 1.0% for the vitrimer containing 50 wt.% of lignin. These lignin-based vitrimers also exhibited excellent performance as recoverable adhesives for different substrates such as aluminium and wood, with a lap shear test strength of 6.0 and 2.6 MPa, respectively. In addition, the preservation of exceeding adhesion performance in recycled lignin-based vitrimer adhesives suggests a promising potential for their application in sustainable circular materials.

## 3. Types of Vitrimers

**Figure 9:**
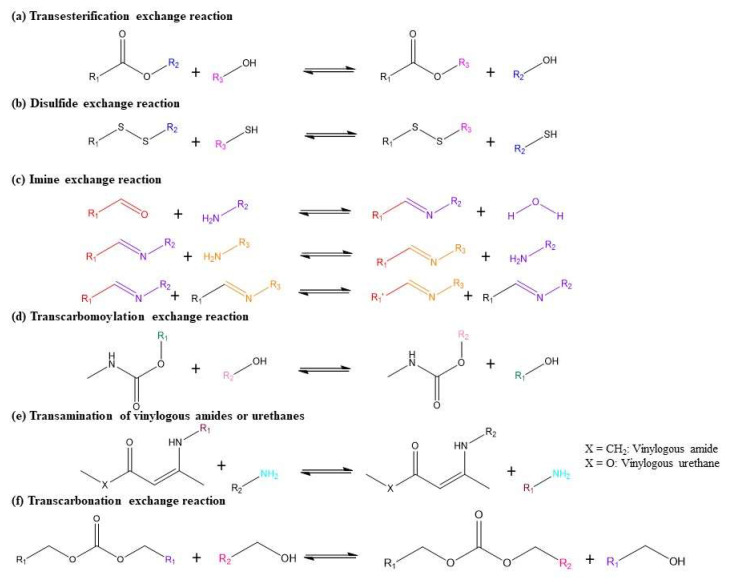
Dynamic chemistries for the synthesis of vitrimer. (**a**) Transesterification, (**b**) disulfide exchange, (**c**) imine exchange, (**d**) transcarbomoylation, (**e**) transamination of vinylogous amides or urethanes, and (**f**) transcarbonation reaction.

To respond to the circular economy and decarbonisation strategy, the application of a synthesis procedure involving “green” feedstocks, safer solvents and reaction conditions is a promising strategy for the development of bio-based vitrimer. Common dynamic exchange reactions to produce vitrimers are presented in Figure 9.

### 3.1. Transesterification Vitrimers

A transesterification reaction is a conversion of a carboxylic acid ester into another carboxylic ester with the presence of an acidic or basic catalyst [55]. The generated ester provides outstanding properties for the resulting materials such as manoeuvrability, adhesion, water resistance, grinding ease, etc. [18], offering great potential for the development of high-performance vitrimer-based CFRCs. An epoxy/acid or epoxy/anhydride polyester polymer network is the first reported vitrimer produced through a transesterification reaction of epoxy and an acid/anhydride and has been extensively studied [17,56]. Specifically, dynamic transesterification-based bio-derived vitrimers are usually prepared by reacting epoxidized vegetable oils (i.e., epoxy-containing compounds) with fatty acids (i.e., carboxyl-containing compounds) to form a polyester which is exchangeable under external stimuli (i.e., heat, light, pH, or pressure, etc.). The general dynamic esterification scheme is illustrated in Figure 9. Based on this feasible reaction mechanism, a number of dynamic ester exchange reactions from natural epoxy-containing compounds have been explored to design eco-friendly vitrimers, responding to the green and sustainable CFRCs goal.

For example, a fully bio-based vitrimer was synthesised through an exchange reaction of rosin-derivative fumaropimaric acid (FPA) and epoxidized soybean oil (ESO) with the presence of zinc acetylacetonate as a transesterification catalyst [14]. The obtained ESO-based vitrimer exhibited a high glass transition temperature (T_g_) of 65 °C and high tensile strength of up to 16 MPa, compared to other ESO-based vitrimer (e.g., ESO/glycidylamine vitrimer showed a T_g_ range of −10–25 °C [57]) (Table 1). Additionally, the vitrimer was found to be easily degraded into precursors using ethanol solvent at 140 °C and reconverted into a new vitrimer at 180 °C. Another study reported a dynamic ester exchange of ESO with natural camphoric acid (CPA) [29]. In this work, a dynamic epoxy matrix was produced by cross-linking ESO with CPA via transesterification in the presence of 1,5,7-triazabicyclo[4.4.0]dec-5-ene (TBD) catalyst. The ESO/CPA vitrimer possessed high cross-linking density and good thermal stability with a T_g_ range of 40–48 °C for different ESO/CPA ratios. Furthermore, ESO/CPA-based composites could be fully recycled under ethylene glycol as a degradation solvent.

The concept of bio-based vitrimer was also introduced into elastomers by Feng et al. [58]. In this study, a vitrimer-like photothermal-induced elastomer was prepared by cross-linking epoxy natural rubber (ENR) with dodecanedioic through a transesterification reaction with aniline trimer as a catalyst. The obtained cross-linked ENR-based vitrimer could change their topology at elevated temperatures, allowing the vitrimers with the ability to be self-healed and reprocessed. Furthermore, the mechanical properties could be maintained up to 88% after three consecutive grinding/hot-pressing cycles (15 MPa pressure at 200 °C for 20 min for each cycle run). Both shape fixing ratio and recovery efficiency were above 95% in the configuration process, leading to potential applications of a vitrimer-based elastomer in the actuator field.

Owing to their naturally abundant availability, lignocellulose-derived compounds (e.g., cellulose, lignin, vanillin, eugenol, etc.) are also potential alternative feedstocks for the synthesis of bio-derived vitrimers. For instance, Liu et al. [59] reacted eugenol-derived epoxy with succinic anhydride with various ratios (1:0.5, 1:0.75, and 1:1) under zinc acetylacetonate catalyst condition to produce a cross-linked epoxy/anhydride vitrimer. It was found that the vitrimer with a eugenol/succinic ratio of 1:0.5 exhibited fast transesterification at 150 °C, improving their thermal reprocessability and chemically recyclability. It was also found that reducing the amount of succinic anhydride in a eugenol/succinic ratio (1:0.75, and 1:1) led to slower stress relaxation rates of the produced vitrimer. Moreover, the study suggested that all eugenol/succinic-based vitrimers could be decomposed in ethanol at 160 °C and reprocessed into new thermosetting polymers at 190 °C, which is comparable to epoxidized soybean-oil-derived vitrimer reported in a previous study [14]. Such a phenomenon was also observed in a lignin-based vitrimer reported by Zhang et al. [33]. A modified Kraft lignin was reacted with sebacic acid in the presence of a zinc catalyst to produce a lignin/sebacic acid polymer network. The resulting vitrimer showed fast stress relaxation, excellent shape memory, self-healing, malleability, and repairing properties at elevated temperatures.

Most reported epoxy vitrimers required catalysts to promote the dynamic transesterification process [60]. Zinc acetate [Zn(OAc)_2_], zin acetylacetonate anhydride [Zn(acac)_2_], tin(II) 2-ethylhexanoate [Sn(Oct)_2_], titanium(IV) isopropoxide [Ti(OPr)_4_], and organic bases such as 1,5,7-triazabicyclo[4.4.0]dec-5-ene (TBD), and 1,8-diazabicyclo[5.4.0]undec-7-ene (DBU), etc., are common transesterification catalysts [24]. However, the utilisation of catalysts can be problematic due to the catalyst leaching during the synthesis process, eventually causing a deactivation of the catalyst as well as the loss in processability of final products [36]. In addition, catalyst-based vitrimers can be limited in their utilisation due to their toxicity, incompatibility, and corrosiveness [26]. Moreover, most of the transesterification catalysts are generally evaporated or decomposed in high heating conditions (>200 °C), limiting long shelf life and commercial applications [24]. Therefore, it is desirable to develop transesterification-derived vitrimers in catalyst-free conditions.

Recently, some efforts have been made to produce bio-based vitrimers in a non-catalyst pathway. Debnath et al. (2020) synthesised a set of catalyst-free vitrimers by reacting a cost-effective natural malonic ester with poly(hydroxyethyl methacrylate) via transesterification reaction at 150 °C without the presence of catalysts. The resulting vitrimers were readily reprocessable and self-healable under thermal conditions with reserved mechanical properties. Furthermore, the study found the reaction rate was increased and the processability temperature was decreased to 100 °C by adding a small amount (5 wt.%) of Sn(Oct)_2_ catalyst. Produced vitrimers from both catalyst and catalyst-free conditions displayed tensile strength of 11.3–33.0 MPa, and elongation at a break of 80–290%. Another catalyst-free epoxy/acid vitrimer was synthesised by curing a vegetable-oil-derived dimer acid (DA) and a N,N,N′,N′-tetraglycidyl-4,4′-diaminodiphenylmethane (TGDDM) epoxy [57]. The formation of a catalyst-free polymer network was found to be promoted by tertiary amines originating from TGDDM, acting as an internal catalyst for transesterification. The resulting network structure contained abundant β-hydroxyester linkages and exhibited a tensile strength of 12.1 MPa and an elongation at a break of 179%, which were comparable to those of commercial polymers and superior to those of vegetable-oil-based polymers reported in previous studies [61,62]. This catalyst-free bio-derived vitrimer also demonstrated unique hydrothermal degradation above 170 °C without using any catalyst, and complete decomposition in water at 190 °C for 5 h.

Inspired by the internal catalyst tertiary amines in a dynamic ester exchange system, a room-temperature curable epoxy vitrimer was prepared by reacting a mixture of hempseed-oil-based epoxy and diglycidyl ether of bisphenol A with diethylenetriamine (DETA) [27]. Under the internal catalytic condition of tertiary amine, producing an epoxy/amine polymer network accounted for up to 67.5 wt.% in the composition and exhibited glass transition temperature (T_g_ > 40 °C), scratch hardness, gouge hardness, adhesive strength, and solvent resistance properties as well as degradability in pure water under tertiary amine catalyst. As the introduction of the tertiary amine is an effective solution for the non-catalyst-required transesterification reaction, Xu et al. [26] synthesised epoxidized menthane diamine-adipic acid vitrimer network via dynamic transesterification reaction of bio-based menthane diamine and adipic acid. The fabricated vitrimer had excellent reprocessing, shape memory, and self-adhesive properties. The scratched vitrimer was completely healed when heated to 180 °C for 60 min. Also, the vitrimer could be fully degraded in ethanolamine solvent at 60 °C for 30 min and catalysed by tertiary amine in the matrix network.

### 3.2. Disulfide Vitrimer

Owing to the ease of synthesis from commercially available starting materials, the short stress relaxation at high temperatures without any addition of catalyst, and the capacity of reprocessing, repairing, and recycling the produced vitrimers, the disulfide exchange mechanism has been extensively studied to produce high-performance vitrimers for CFRCs applications [22,35,63,64].

Ma et al. [65] investigated a bio-based vitrimer produced by reacting an isosorbide-derived epoxy with a disulfide-containing aromatic diamine via dynamic disulfide exchange. Particularly, the homogeneous mixture of isosorbide-based epoxy and 4-aminophenyl disulfide was moulded and cured at 80 °C for 1 h then 150 °C for another hour. The obtained vitrimers possessed distinguished properties such as an easy preparation process from cost-effective naturally occurring epoxy monomer and readily available aromatic disulfide, with a fast stress relaxation time at moderate temperatures under catalyst-free conditions, and degradation in alkaline adequate solution (NaOH) instead of organic solvents which was greatly beneficial to the environmental conservation. Nonetheless, the recovery efficiency was found to be gradually decreased after repeated grinding/hot pressing, which was due to the possible oxidation of sulfur radicals at high heating conditions or the scission of C-C bonds during the grinding process.

For the utilisation of vegetable oils in disulfide exchange-based vitrimers, Mauro et al. [63] designed a recyclable, repairable, and reshapeable (3R) thermosetting resin by copolymerising twelve different epoxidized vegetable oils (EVO) with 2,2′-dithiobenzoic acid as a hardener via dynamic disulfide exchange reaction. It was the first work exploiting a large number of EVOs in a vitrimer system. These oils were categorised into three groups based on their nature and unsaturated fatty acid content. Mono-unsaturated fatty acid compounds (group I) contained karanja, castor, St John’s Wort, peanut, and rapeseed oil. Di-unsaturated fatty acid compounds (group II) contained soybean, rosehip seed, safflower, grapeseed, and hemp oil. Group III were tri-unsaturated fatty acid compounds composed of camelina, linseed, and perilla oil. It was found that oil-based vitrimers from group I possessed low epoxy content due to a lower degree of cross-linking nodes, correlating to lower glass transition temperature and mechanical properties, but showing greater performance of reprocessability and better solvent resistance compared to the vitrimers from the other groups. The properties of these EVO-based vitrimers are shown in Table 1.

Mauro and the team also investigated the influence of imidazole initiator on the performance and the reprocessing properties of the resulting vitrimers [64]. Particularly, an epoxy/diacid polymer network was synthesised by reacting a vegetable-oil-based epoxide with an aromatic diacid containing disulfide bonds through a dual mechanism of disulfide metathesis and transesterification. The produced vitrimer-like thermosets showed very good reprocessability and full recyclability in 1 M NaOH at 80 °C for 3 days without any addition of catalysts. Additionally, the functionalisation of the recycled thermosets was not significantly changed even after 10 reprocessing cycles.

### 3.3. Polyimine Vitrimer

A polyimine dynamic bond, also known as a Schiff base, is currently drawing great interest in developing high-performance vitrimers owing to their excellent flexibility and fast cross-link exchange without a catalyst. Particularly, imine bonds can be easily formed by the condensation reaction of primary amines and aldehydes or ketones, as shown in Figure 9. Additionally, imine bonds can undergo a transamination reaction with another primary amine and an imine metathesis reaction with another imine bond [28].

Recently, Liu et al. [25] prepared a bio-based epoxy vitrimer with dynamic polyimine cross-links from epoxidized soybean oil, vanillin and 4-aminophenol. In particular, vanillin reacted with 4-aminophenol in ethanol/water solvent to form a mixture acting as a dynamic curing agent. The mixture was then cured with epoxidized soybean oil at a curing agent/epoxy equivalent ratio of 0.7/1.0 with 1,2-dimethylimidazole as a catalyst. The resulting vanillin-based epoxy vitrimer showed promising malleability, recyclability, and mechanical properties, making it a potential matrix to fabricate carbon fibre composites. Additionally, the produced vitrimer-based CFRC (once crushed) could be recycled through thermal processing, resulting in short carbon fibres which may have the potential for non-structural applications. In another study, vanillin reacted with hexachlorocyclotriphosphozene via nucleophilic aromatic substitution reaction to produce a hexasubstituted cyclotriphosphazene containing multiple aldehyde groups. This aldehyde-containing compound was then reacted with different diamines to form reversible polyimine bonds through a Schiff base reaction. The polyimine vitrimers cured with 1,2-bis(2-aminoethoxy)ethane showed a tensile strength of 57.32 MPa, and elongation at a break of 9.73%, whereas the vitrimer cured with ethylenediamine exhibited the T_g_ at 119.37 °C. Additionally, these vitrimers performed excellent solvent resistance in an alkaline solvent, natural solution brine, ethanol, and water, but could be degraded in an acidic solution to recover up to 95 wt.% of the monomers [66].

However, compared to industry-grade epoxy resin, biobased polyimine vitrimers still show inadequate mechanical properties and limited water sensitivity, which restricts their applications in CFRCs. Furthermore, polyimine vitrimers are susceptible to creep due to their highly active cross-link exchange at ambient temperature, making them unsuitable for applications that require high dimensional stability [67]. Recently, the integration of metal complexes has been applied to address the poor dimensional stability of polyimine vitrimers. Wang and the team [32] introduced three different metal complexes (Cu^2+^, Fe^3+^, Mg^2+^) into a polyimine vitrimer through a one-pot procedure involving the formation of metal complexes and cross-linking of polyimine. It was found that the increasing addition of Cu^2+^ content enhanced the creep resistance of the prepared vitrimers. Specifically, the increased loading of Cu^2+^ (mol %) raised the initial creep temperature from 60 °C (with 0.5 mol % Cu^2+^) to 100 °C (with 5 mol % Cu^2+^) and improved the Arrhenius activation energy for stress relaxation from 52.3 to 67.7 kJmol^−1^. The effectiveness of different metal complexes on the improvement of creep resistance followed the order of Fe^3+^ > Cu^2+^ > Mg^2+^. The resulting vitrimers showed enhanced thermal and mechanical properties and acid resistance. However, water resistance of these vitrimers was relatively poor, which was attributed to the poor stability of metal coordination bonds in water. To enhance the water insensitivity of the vitrimer, Liu et al. [28] synthesised a bio-based vitrimer by curing lignin-derived vanillin with m-xylylenediamine under dynamic imine exchange reaction. The obtained vitrimers showed improved water insensitivity that was not observed in other polyimine-based vitrimers. In particular, these vitrimers achieved a tensile strength (70.5 MPa) and a Young’s modulus of 2.32 GPa at a breaking elongation of 6.67% after being immersed in water for 15 days. Additionally, the vitrimer materials exhibited short stress relaxation behaviour (16s at the temperature of 40 °C above T_g_), adequate self-healing, weldability, and remouldability properties, etc. Moreover, degradation and eco-friendly recycling capabilities under mildly acidic solvents were also observed in this polyimine-based vitrimer. A previous study also reported performance-modified polyimine vitrimers obtained from vanillin and aromatic diamine (m-xylenediamine dimer) [68]. The resulting vitrimers possessed flexibility at ambient temperature, excellent thermal stability (a mass loss of 6.3% at 300 °C), and short relaxation time (from 260 s at 70 °C to 22 s at 100 °C). In addition, the obtained networks also showed their reprocessability and recyclability in a short time and lower temperature under the conditions of heat, water, and amine solvents without any addition of specific catalysts.

Fast stress relaxation behaviour is one of the important properties of vitrimer in facilitating the recyclability and reprocessability of vitrimer-based composite materials [69,70]. Particularly, stress relaxation is a reduction in stress in response to strain over time [71]. The relaxation time is mostly modified by chemical controls such as choice of dynamic linkages, catalyst types and amounts, the effect of side groups on chemical structure, etc. [72,73]. On the other hand, to control the relaxation property of vitrimers by physical controls, Hajj and their team [70] reported the impact of the cross-link density of the polymer network on relaxation time. Specifically, the vitrimers were synthesised from furan-derived dialdehyde and di- or tri-functional polyetheramines via the polyimine dynamic exchange reaction. The thermal stability, tunable mechanical properties, and reprocessability of the obtained polyimine vitrimer were evaluated, although the process still offered robustness against aggressive conditions such as the use of a highly basic solvent (1M NaOH). However, the vitrimers were completely dissolved in an acidic solution (1M HCl) due to the acid-catalysed hydrolysis of imine bonds [70]. The cross-link density has a significant impact on the relaxation behaviour of the resulting vitrimers, which could be reduced 20-fold with a decrease in the cross-link density [70]. Another typical property of vitrimers is their self-healing ability. This feature, often associated with reprocessing, is enabled by introducing exchangeable cross-links into the polymer networks. The covalent adaptable networks in vitrimers require a relatively high temperature (>150 °C) to preserve their integrity, which would restrict their applications and increase the fairly high cost of recovering [45,74]. A recent study has introduced renewable vanillin into a solvent-induced recyclable polyimine vitrimer, whose self-healing and reprocessing could be carried out both in traditional hot pressing and in a fast gel–sol transition under milder temperature conditions [74].

### 3.4. Other Dynamic Chemistries

The transcarbonation exchange reaction occurs when a hydroxyl nucleophile reacts with a carbonate to form an associative intermediate and releases exchanged carbonate and hydroxyl groups [75] (Figure 9f). For example, a catalyst-based carbonation exchange reaction was investigated for polycarbonate vitrimers by Snyder and co-workers [16]. In this study, a hydroxyl-functionalised polycarbonate network was prepared by reacting 1,4-butanediol with bis(6-membered cyclic carbonate) at elevated temperature in the presence of a simple and inexpensive Titanium (IV) isopropoxide catalyst. The obtained polymer networks showed high recovery of their tensile strength and storage modulus (71–133%). In addition, the vitrimer networks could be hydrolysed and decarboxylated in an acidic solvent to recover up to 80 wt.% of the precursors. Moreover, polycarbonates from transcarbonation reactions have great potential for the development of sustainable vitrimer productions because the starting materials can be derived from renewable bio-based resources. For instance, hydroxyl groups can be synthesised from vegetable oils, fatty acids, lignocellulose, etc., and carbonates can be obtained from carbon dioxide [76,77].

Another dynamic reaction pathway for the preparation of vitrimer is the transamination of vinylogous acyls, which is a competitive mechanism for transesterification [78]. Vinylogous transamination is a catalyst-free condensation reaction of β-ketoester and primary amines. This dynamic chemistry has been extensively applied to produce vitrimers owing to its easy installation of acetoacetate and amine compounds [42]. As the name implies, this mechanism involves free amine groups, whose stability is vital to preventing long-term oxidative damage [78]. The first vinologous urethane vitrimers produced from a transamination reaction were reported by Denissen and co-workers [79]. The obtained poly(vinylogous urethane) vitrimer networks showed a good storage modulus of 2.4 GPa, glass transition temperature above 80 °C, short relaxation time of 85 s at 170 °C, without the presence of any catalyst. Additionally, the vitrimer could be recycled up to four times by consecutive grinding or compression moulding cycles without significant loss of mechanical and thermal properties. Particularly, no change was observed in the Young’s modulus and stress at break, while strain at break ranged from 5.5 to 7.5%. With the growing interest in employing natural resources as raw materials to produce eco-friendly vitrimers, Zhu et al. [42] developed a fully bio-based vinylogous urethane vitrimer using renewable castor oil and DL-limonene. The prepared vitrimer showed excellent shape memory and good mechanical properties (Young’s modulus of 27.2 ± 1.9 MPa, stress at break of 5.5 ± 0.3 MPa, and elongation at break of 126 ± 5.5%). Moreover, this vitrimer possessed higher T_g_ (39 °C) than other reported vinylogous vitrimers [69,80]. Recently, Hajiali et al. [10] reported a novel bio-based vitrimer via the reaction between β-ketoesters (derived from 2-acetoacetoxy ethyl methcrylate and isobornyl methacrylate) and bi-functional amine (derived from vegetable oils). The T_g_ of the obtained vitrimers was improved from 15 to 90 °C by increasing isobornyl methacrylate from 10 mol% to 90 mol%. The vinylogous vitrimers also exhibited high recyclability by grinding and remoulding at 125 °C without mechanical degradations.

A transamidation-based vitrimer from natural resources was introduced by Pettazzoni and co-workers [81]. In this work, the vitrimer was developed through the cross-linking of a renewable monomer (methyl oleate epoxidized) with tris(2-aminoethyl)amine in the presence of boric acid as a green, cheap and non-toxic catalyst for transamidation reaction. The resulting vitrimers showed good thermal stability up to 300 °C and the T_g_ value ranging between 7 and 21 °C, depending on the amount of acid catalyst.

**Table 1:** Overview of bio-based vitrimers including their mechanism, glass transition temperature, strength, and degradation properties.

Materials	Mechanism	Properties				References	Cost Estimation ^a^
		Glass Transition Temperature (Tg)	Tensile Strength (MPa)	Young’s Modulus (GPa)	Recycling Condition		
ESO and rosin-based fumaropimaric acid	Transesterification	65	16	-	Ethanol solvent at 140 °C	[14]	285–292 AUD/kg
ESO and camphoric acid		40–48	0.56	0.0024	Ethylene glycol at 190 °C	[29]	986 AUD/kg
Eugenol-derived epoxy and succinic acid		42–47	-	-	Ethanol at 160 °C	[59]	1763–2030 AUD/kg
Malonic ester and poly(hydroxyethyl)		-	11.3–33.0	0.317–1.112	-	[24]	900 AUD/kg
Vegetable-oil-based dimer acid and glycidylamine		−10–25	12–22	0.014–0.037	Hydrothermal at 90 °C	[57]	453–550 AUD/kg
Hempseed-oil-based epoxy and diglycidyl bisphenol A		40	54–75	1.02–1.29	-	[27]	397 AUD/kg
Adipic acid and methane diamine		72–86	57–75	1.9–2.3	Ethanolamine at 60 °C	[26]	1236–1436 AUD/kg
Isosorbide-derived epoxy and 4,4′-methylenedianiline	Disulfide exchange	37.3	11.37	2.36	5 wt.% NaOH solution	[65]	169 AUD/kg
Isosorbide-derived epoxy and 4,4′-disulfanediyldianiline		41.4	10.98	1.99			9110 AUD/kg
EVO—mono-unsaturated fatty acids and 2,2′-dithiodibenzoic acid		17–40	-	-	Mechanical recycling: grounded vitrimer powder was pressed and heated (120–170 °C; 10 min, 1–2 tons depending on fatty acid types)	[63]	510–545 AUD/kg ^a^*
EVO—di-unsaturated fatty acids and 2,2′-dithiodibenzoic acid		45–62					
EVO—tri-unsaturated fatty acids and 2,2′-dithiodibenzoic acid		64–91					
ESO and vanillin	Polyimine exchange	27.6	7.7	0.04	HCl at RT	[25]	436 AUD/kg
Glycerol triglycidyl ether and vanillin		70	62	1.62	Amine solution at RT	[82]	402 AUD/kg
Vanillin and 4,4′-diaminodiphenylmethane		102	80.3	2.89	HCl at 60 °C	[83]	744 AUD/kg
Vanillin and isophorondiamine		99	53.9	1.56			774 AUD/kg
Vanillin and diethyltriamine		66	33.5	1.39			814 AUD/kg
Vanillin and 1,6-hexylenediamine		83	73.2	1.96	HCl at RT	[84]	279 AUD/kg
Vanillin and m-xylenediamine		96	78.3	2.39			299 AUD/kg
Castor oil and DL-limonene	Vinylogous urethane	39	5.5	0.027	-	[42]	25678 ADU/kg
β-ketoesters and vegetable-oil-based amine		15–90	5.12	0.096	Amine solution	[10]	283 AUD/kg
Methyloleate epoxy and tris(2-aminoethyl)amine	Transamidation	7–21	1.5	0.017	-	[81]	5062 AUD/kg

^a^ Cost estimation is based on price of raw materials (with an equivalent weight ratio required for the synthesis) from Sigma-Aldrich. Details can be found in the supporting document; ^a^* Cost is based on epoxidized soybean and linseed oils.

## 4. Bio-Vitrimer-Based Carbon Fibre Composite

### 4.1. Use of Bio-Based Vitrimer Matrix for Sustainable CFRCs

**Figure 10:**
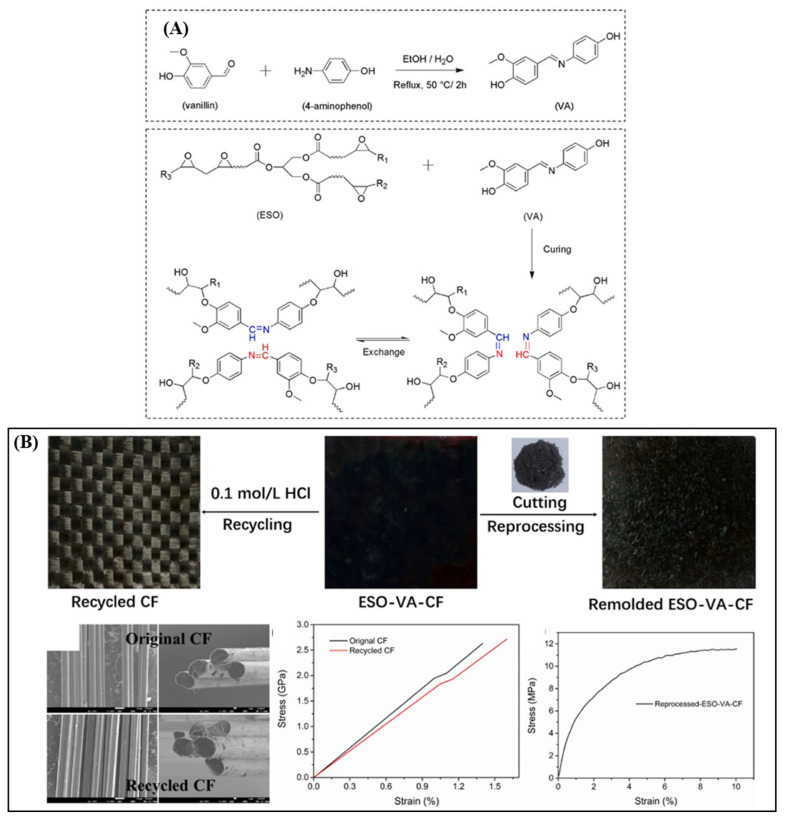
(**A**) Synthesis of vanillin-based epoxy vitrimer, and (**B**) Comparison in tensile strength between the original and recycled CF. Adapted with permission from [25].

Recent attempts have been made to explore and report the potential utilisation of bio-based vitrimer in reinforced composites with significant achievements. For example, Liu et al. [25] cured the dynamic vanillin-based imine-containing agent with epoxidized soybean oil to produce epoxy vitrimer as a matrix for carbon fibre composites (Figure 10A). The soybean-oil-derived composite showed a tensile strength of 145.4 MPa and a modulus of 1.18 GPa. In addition, the carbon fibre could be completely recycled without damaging structure and properties. The short carbon fibre once crushed and recycled through thermal processing could also have potential for non-structural applications (Figure 10B). This team also reported another type of imine-containing curing agent from vanillin, 4-aminophenol, and glycerol triglycidyl ether, which was used as a matrix for carbon fibre materials [82]. The obtained composite exhibited a tensile strength of 449 MPa, Young’s modulus of 12.9 GPa, and elongation at a break of 4.6% (Tabel 2). Additionally, the composite could be fully degraded in an amine solution. The recycled products (non-destructive carbon fibres and vitrimer resins) could be sufficiently reused to prepare the secondary application composite, which paved the way for a full recycling process for carbon CFRCs.

**Figure 11:**
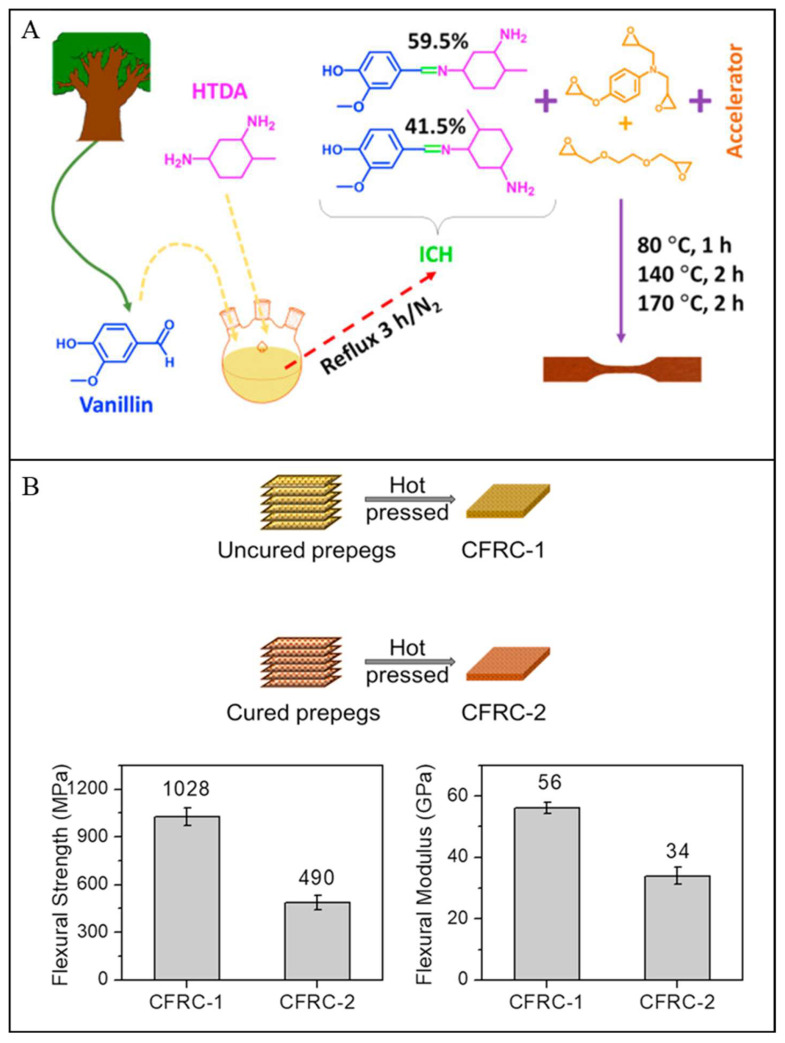
(**A**) Synthesis of vanillin-based epoxy vitrimer, and (**B**) Fabrication and flexural strength of CFRCs. Adapted with permission from [3].

Another bio-based imine-containing epoxy vitrimer used as a matrix for carbon fibre composite was developed by Memon and co-workers (Figure 11) [3]. The epoxy vitrimer was found to have sufficient T_g_ (≥131 °C), tensile strength (≥82 MPa), and solvent resistance. The vitrimer-based composite was fabricated by a conventional hot-pressing technique using uncured vitrimer-based carbon fibre prepregs, which were soft and tacky as typical thermosetting prepregs. The resulting composites showed good flexural strength (1028 MPa) and modulus (56 GPa) which were comparable to the reported values from conventional thermosetting prepregs. However, the composite prepared by fully cured prepregs showed reduced flexural strength (490 MPa) and modulus (34 GPa), which were 47 and 60% of the composites produced from uncured prepregs. This was due to the existence of carbon fibres, which prevented the epoxy vitrimers from close contact and effective bond exchange. Additionally, it was found that the viscosity of the vitrimer matrix during the hot press was still high, hindering the diffusion of the matrix into the carbon fibres. Although the composite from cured prepregs showed a reduction in mechanical properties, they exhibited high reparability, where 92% strength recovery was obtained for the recovered CFRCs. Dynamic imine exchange bonds in a bio-based vitrimer system was also reported by Wang et al. [83]. In this study, a vanillin-based dialdehyde monomer and commercially available amines were used to prepare a polyimine vitrimer. The tensile strength and Young’s modulus of the obtained polyimine vitrimer reached 80.3 MPa and 2.89 GPa, respectively. Additionally, the two-layer carbon fibre composite exhibited a tensile strength of 505.4 MPa and a Young’s modulus of 5.52 GPa, which, however, was slightly inferior to that produced from petroleum-based bisphenol A epoxy due to the relatively poor interface. Owing to the imine chemistry, the vitrimer was easily degraded under an acidic solution without affecting the surface morphology and chemical structure of the recovered carbon fibres.

**Figure 12:**
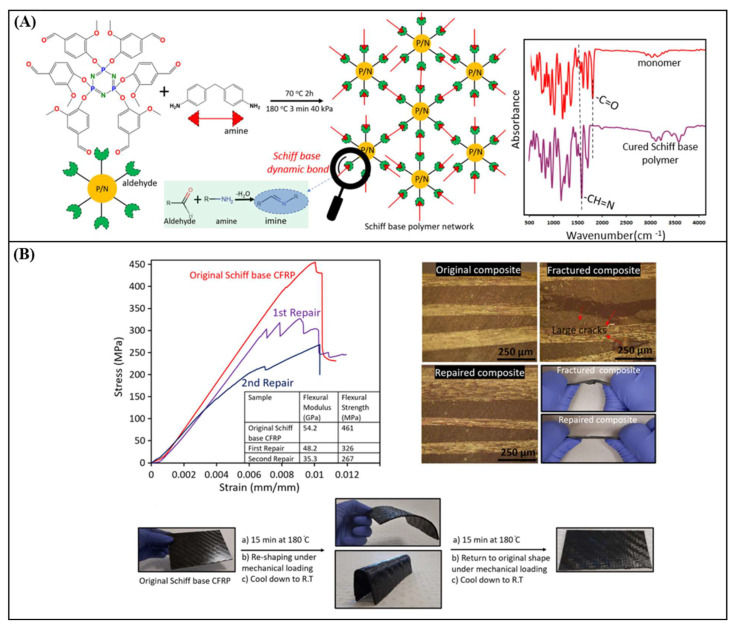
(**A**) Synthesis of vanillin-based Schiff base vitrimer, and (**B**) Flexural properties of original Schiff base CFRC with two-time repaired CFRC. Adapted with permission from [85].

In another study, vanillin was used to synthesise a multifunctional Schiff base monomer containing six aldehyde reactive sites, which was cured with a diamine to form a highly cross-linked Schiff base vitrimer (Figure 12) [85]. The obtained vitrimer was then used to fabricate the Schiff base CFRC. It was found that the Schiff base CFRC exhibited high tensile (461 MPa) and flexural strength (455 MPa), with a relatively high glass transition temperature (129 °C) (Table 2). Additionally, compared to the CFRC obtained from industrial epoxy grade, the Schiff base CFRC showed good repairability and thermoformability under initial processing conditions, and retained ~70 and ~58% of its original flexural strength after reprocessing two times (Figure 12B). Moreover, owing to the presence of nitrogen/phosphorus rings in their chemical structure (Figure 12A), the obtained CFRCs had outstanding fire retardancy, showing a V0 rating in the UL94 test.

**Figure 13:**
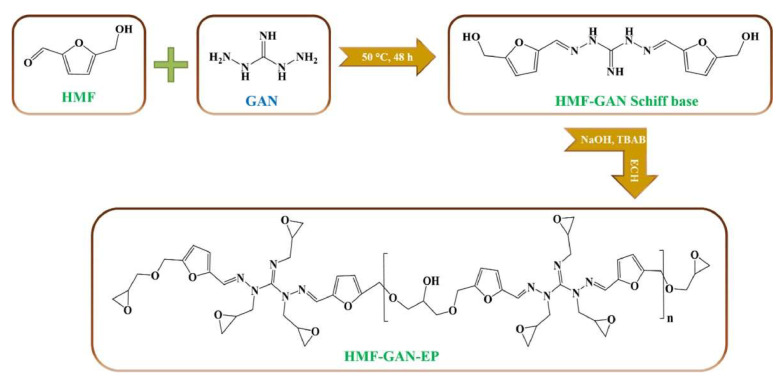
Synthetic route for furan-based vitrimer. Adapted with permission from [86].

Research by Nabipour and their team also focused on the flame retardancy of CFRC [86] (Figure 13). In this study, a Schiff base vitrimer was produced from 5-hydroxymethylfurfural (HMF) and 1,3-diaminoguanidine monohydrochloride (GAN) and was then cured with a furan-based curing agent (DIFFA) to form a vitrimer system with outstanding flame retardancy (UL-94 V-0 and LOI of 40%). It was also found that the vitrimer could be recycled by dissolving it in a mixed solution of THF:HCl (8:2, *v*/*v*) for the vitrimer to undergo the amine–imine exchange reaction, which, in turn, converts them back to the monomers.

A bio-based catalyst-free vitrimer from dynamic transesterification and its use as a polymer matrix for the composite was reported by Liu et al. [87]. In particular, maleic anhydride and glycerin were thoroughly mixed with itaconic acid-based epoxy resin and cured without any solvent or catalyst. The preparation of vitrimer-based composites was conducted using a vacuum infusion process. The resulting composites showed moderate tensile strength (417 MPa) and modulus (31.3 GPa). Additionally, these composites showed outstanding degradability in sodium hydroxide with mild conditions. The monofilament tensile strength of the first, second, and third recovered carbon fibre retained 98%, 97%, and 94% of the virgin carbon fibre, respectively. The multiple recycling of carbon fibre with an almost non-impaired structure would bring a potential economic benefit considering the high cost of carbon fibres. Recently, a catalyst-free bio-based vitrimer matrix obtained from a degradable epoxidized menthane diamine and adipic acid through a dynamic transesterification reaction was used to prepare a vitrimer-based composite [26]. The composite can be fully degraded in ethanolamine solution at 60 °C for 30 min. The chemical structure and mechanical performance of the recovered carbon fibre were almost unaffected. However, the recycling process occurred at a relatively low temperature, which might limit the utilisation of this material in some high-temperature service applications.

**Table 2:** Thermal and mechanical properties, and recyclability of the bio-based vitrimer CFRCs.

Samples	T_g_ (°C)	T_d5%_ (°C)	Tensile Strength (MPa)	Young’s Modulus (GPa)	Flexural Strength (MPa) and Modulus (GPa)	Recycling Condition	Reference
Epoxidized soybean oil & vanillin & 4-aminophenol	27.6	-	145.4 ± 17.13	1.18 ± 0.14	-	0.1 M HCl for 20 h for the resin matrix to be completely dissolved.	[25]
Vanillin & 4-aminophenol & glycerol triglycidyl ether	70	257	449 ± 12	12.9 ± 0.9	-	0.1 molL^−1^ ethylene-diamine (EDA)	[82]
Vanillin & methylcyclohexane-diamine	>131	267–284	-	-	>490 & >34	EDA, with epoxy resin:EDA ratio was 1:5	[3]
Vanillin & phosphonitrilic chloride trimer & 4,4-diaminodiphenyl methane	129	215	461 ± 21	47.3 ± 2	455 ± 14 & 54.2 ± 4	THF/HCl at RT for 24h	[85]
Hyperbranched polyesters (HBP) & rosin-derived fumaropimaric acid & glycerol triglycidyl ether	75.6–90.5	-	585 ± 35 (with the 15 wt.% of HBP in the vitrimer system	-	-	Ethylene glycol (EG) at 180 °C for 12 h under N_2_ atmosphere	[88]
5-hydroxymethylfurfural (HMF) & 1,3-diaminoguanidine monohydrochloride (GAN) & furan-based curing agent (DIFFA)	234.1	-	-	-	-	THF:HCl (8:2, *v*/*v*)	[86]
Itaconic acid & maleic anhydride & glycerine	49–56	285	417 ± 26	31.3 ± 1.8	-	1 M NaOH at RT for 5 h	[87]
Adipic acid & epoxidized menthane diamine	72.1–86.4	282 (for T_d10%)_	465	-	-	Ethanolamine at 60 °C for 30 min	[26]

### 4.2. Processability of Vitrimers by Their Flowable and Thermoformable Behaviours

One of the important features of vitrimers is thermoforming in which the dynamic bonds can be redistributed through the vitrimer system upon heating while maintaining the cross-linked density. This leads to a lower viscosity at elevated temperatures, and ultimately malleability of the vitrimer network. At high temperatures, the exchange reaction becomes fast enough to reduce the viscosity of the vitrimer which follows the Arrhenius law. Thus, a study on the viscosity behaviour of vitrimers is necessary to investigate the processability of this material. Specifically, to flow the vitrimer, cross-linked networks should be able to reorganise their topology at elevated temperatures but below degradation. The temperature at which the topology of the vitrimer network changes in response to temperature is called the topology freezing temperature (T_v_). Therefore, the study of the flow behaviour of the vitrimer also includes the T_v_, in addition to the T_g_ [89]. The T_v_ is related to the freezing of network topology due to the absence of exchange reaction on the timescale of the observation [90]. Since the vitrimer was explored in 2011 by Leibler’s group, the hypothetical T_v_ is chosen as the temperature at which the melt viscosity reaches 10^12^ Pa s [15].

**Figure 14:**
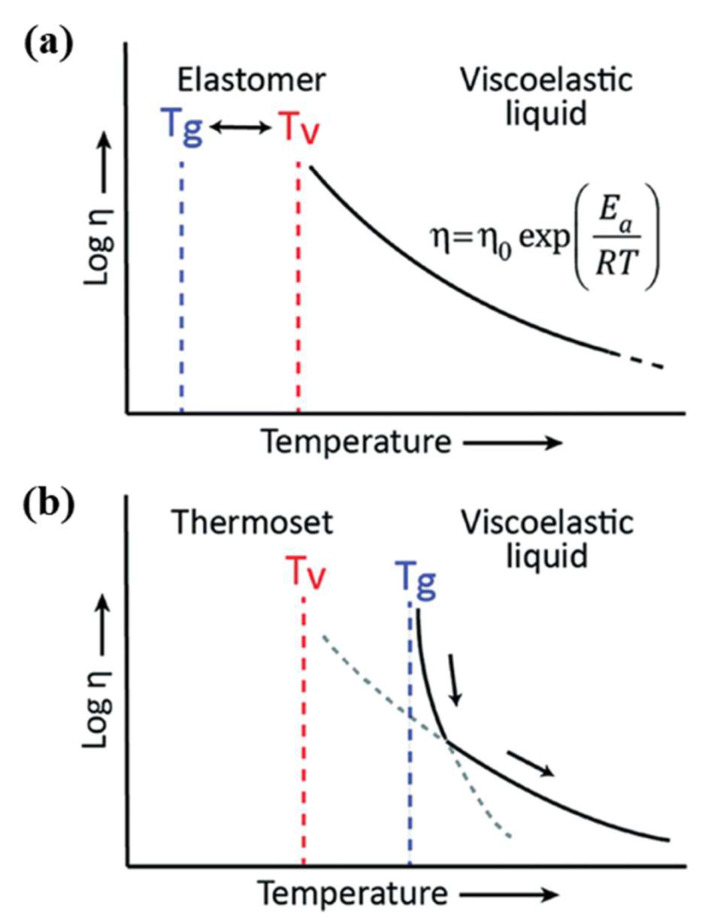
Viscoelastic behaviour of vitrimer where (**a**) the glass transition temperature, T_g_, is greater than the topology freezing temperature, T_v_; and (**b**) T_g_ < T_v._ Adapted with permission from [89].

Above the T_v_, the vitrimer exhibits viscous flow due to the faster exchange reaction. On the other hand, below the T_v_, it behaves as a conventional thermoset or elastomer because the kinetic exchange reaction is very slow, hence “freezing” the network topology. The relationship between T_v_ and T_g_ is shown in Figure 14. In the case of T_v_ > T_g_ (Figure 14A), when it is heated above its T_g_, the vitrimer undergoes the transition from a glassy solid to an elastomer because the network topology is still fixed (i.e., the exchange reaction rate at this stage is very slow). Upon further heating to reach the T_v_, the exchange reaction increases and becomes fast enough to allow macroscopic flow, and the material behaves as a viscoelastic liquid at this stage, with a melt viscosity gradually decreasing, which follows the Arrhenius law [89,90]. In the case of T_v_ < T_g_ (Figure 14B), the fast exchange reaction above T_v_ is trapped in a rigid glassy matrix, preventing the topological rearrangement. When the temperature reaches T_g_, the viscosity starts to drop and first follows the William–Landel–Ferrt (WLF) model due to the initiation of segmental motion and then follows the Arrhenius model when the topological reconfiguration becomes predominant [89].

**Figure 15:**
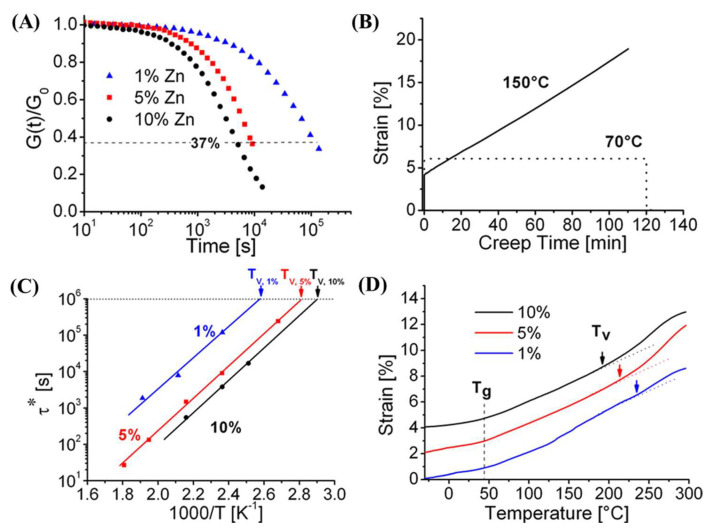
(**A**) Stress–relaxation curves for 1, 5, and 10% catalyst, (**B**) elongation creep curve for 10% catalyst, (**C**) Arrhenius plot of measured relaxation times (τ*), and (**D**) DSC curves for 1, 5, and 10% catalyst. Adapted with permission from [92].

The viscoelastic behaviour of vitrimer is usually examined by stress relaxation and creep recovery experiments. Stress relaxation is a typical method to measure the viscoelastic property of vitrimer. Thus, controlling the relaxation behaviour of vitrimer is one of the key points in the development of this material. More specifically, reducing the relaxation time is important to facilitate their recyclability and to allow their processability via common manufacturing techniques such as extrusion, or injection moulding [70]. The control of vitrimer’s relaxation behaviour mainly relies on the catalyst effect and the cross-link density [91]. For example, on the effect of catalyst, Leibler’s team examined the influences of different catalyst concentrations on the flow activation energy in the transesterification-based vitrimer. Specifically, the vitrimer was synthesised by reacting DGEBA with a mixture of di-carboxylic acid and tri-carboxylic acid, in the presence of 1, 5, and 10 mol% of zinc acetyl acetonate catalyst. The study indicated that although the polymer network was insoluble, it could perform a complete stress relaxation at high temperatures and flows. The increasing addition of catalyst leads to a faster exchange reaction, a lower transition temperature, and a lower viscosity, as illustrated in Figure 15.

Another research also investigated the effect of cross-link density on the relaxation behaviour of the vitrimers [70]. This study synthesised the vitrimer by reacting furan-based dialdehyde with a mixture of diamine and triamine, using THF as solvent. The resulting vitrimer network with different cross-link densities was then used to examine their influences on the viscoelastic properties. It was found that the decrease in cross-link density results in a strong reduction in the relaxation time, which is due to the increase in chain mobility within the less cross-linked structure, in response to elevated temperature. This study also indicated the contradiction in vitrimer design, which was that reducing the stress relaxation time improves the processability and recyclability; nonetheless, it leads to lower glass transition and higher creep deformation, causing a severe drawback for industrial applications [70]. In addition to the processability of vitrimer and vitrimer–CFRC, a measurement of the viscosity of vitrimer–CFRC has also been investigated to rate its thermoformability. It should be noted that the viscosity measurement for the vitrimer–CFRC should be considered as a guide for its thermoformability (i.e., re-processability, and thermal recyclability) and does not represent a real viscosity value of the vitrimer matrix. The rheology test is usually performed by the torsion clamp rheometer due to the limitations of the solid materials on the plate rheometer. The thermoformability of the vitrimer–CFRC is also investigated using a tempered three-point bending test. Both tests were studied by Weidmann and their team [93]. This study concluded that the thermoforming behaviour of vitrimer–CFRC is still challenging and needs different process conditions compared to the thermoplastic CFRC due to the high viscosity of the vitrimer matrix even at elevated temperatures above its T_g_. Hence, a deep understanding of the viscoelastic behaviour of the vitrimer, as well as the method (physical and chemical approaches) to tune this property, is necessary to enable high-volume production in industries [70,93].

## 5. Conclusions and Future Perspectives

Enhancing the sustainability of polymer networks is a significant challenge in the polymer industry that needs to be addressed. Hence, researchers are currently investigating the use of bio-based materials to reduce the reliance on non-renewable resources. Although bio-based resources have proven to be potential feedstocks to produce sustainable biopolymers, the field of bio-based vitrimers still remains challenging, and that limits their utilization in advanced applications. Major obstacles as shown below will have to be overcome before the academic research on bio-based vitrimers can be implemented into real-world applications and enable them to shift towards sustainable industrialization.

- Properties: Keeping biobased material performance comparative to commercial synthetic products remains an open question. Many reported biobased vitrimers often exhibit reduced thermal stability and mechanical properties (e.g., tensile strength, flexural strength, hardness, etc.). Indeed, in many studies, bio-based vitrimers showed relatively low T_g_ due to the presence of vegetable oil monomers that often have low thermal stability, resulting in reduced mechanical properties. Therefore, most reported bio-based vitrimers have not yet reached sufficient standards to meet the requirements of the market.
- Durability: Limited solvent resistance is another crucial challenge for the development of bio-based vitrimers. For example, many polyimine-based vitrimers exhibit low acid and water resistance due to the imine chemistry in their structure, restricting their application in harsh conditions.
- Processing: Compared to traditional polymer matrix composites, most vitrimers and biobased vitrimers have unique processing requirements depending on their dynamic chemistry, which, in turn, limits their widespread application in CFRC manufacturing.
- Scalability: Industrial scale is another big challenge for biobased vitrimers, as the current production methods are typically not suitable for large-scale industrial applications.
- Cost: Biobased vitrimers are still more expensive than conventional polymer matrix composites, which is mostly related to the pre-treatment process of the raw bio-resources to obtain the bio-based monomer. Indeed, due to complex and heterogeneous structures, most bio-based monomers are still obtained from multiple modifications involving petrochemical reagents and produce large by-product waste. An example of this is natural aromatic compounds (e.g., vanillin, eugenol, etc.) obtained from the modification of lignin macromolecules and/or from biomass pyrolysis pre-treatments, which is an energy-intensive process.
- Environmental impact: In most studies reported in this review or elsewhere, vitrimers are only partially bio-based, and often require copolymerization with petroleum-based monomers or involve toxic and non-natural catalysts/solvents. For instance, the number of bio-based vitrimers is limited by the availability of bio-based diamines and/or aromatic diamines, leading to the reliance on petroleum-based amines (e.g., diethylenetriamine, tris(2-aminoethyl)amine, etc.). Additionally, many reported bio-based vitrimers obtained from epoxidized vegetable oils through transesterification reactions often involve diglycidyl ether of the bisphenol A monomer, which is very toxic for all living things. This also weakens the sustainability of these bio-based materials.

The outlook of employing recyclable and bio-derived vitrimers is very attractive, posing a unique solution to address the unsustainability and lack of recyclability of thermosetting materials. Over the past few decades, bio-based resources have been considered excellent alternatives to the finite fossil feedstocks, which are rapidly depleting and causing significant environmental issues. Some bio-based materials such as vegetable oils and their derivatives (e.g., dimers of fatty acid, etc.) have been extensively studied for the development of bio-based vitrimers due to their wide abundance, nontoxicity, renewability, and potential for chemical modifications. Naturally aromatic monomers such as carbohydrates, lignin and its derivatives (e.g., vanillin, furan, etc.) are also very attractive to enhance the thermal and mechanical properties of bio-based vitrimers. In addition, as a hydrophobic polymer, lignin may be a potential co-monomer to enhance the water insensitivity of polyimine vitrimers. In general, bio-based vitrimers can be promising in green polymer industries when factors such as availability at low prices, easy modifications/pre-treatments, and their true sustainability are carefully considered. However, the currently available literature regarding the potential utilisation of bio-based vitrimers on carbon fibre composite materials is limited, leaving open doors and plenty of opportunities for future development.

This review article provides comprehensive insights into the current developments and challenges of biobased vitrimer materials for CFRCs. The investigation of bio-based vitrimers represents a promising solution to address the lack of sustainability and recyclability of materials used in CFRC productions. Although having potential recyclability due to the covalent adaptable networks, most bio-based vitrimers reported in this review or elsewhere exhibited low durability and reduced thermal and mechanical properties, which then limit their real-life applications in industry. For example, many of the soybean-oil-based vitrimers from the transesterification mechanism showed a glass transition temperature (27–65 °C) that is too low for the desired composite application temperature ranges. One such pathway to produce a vitrimer with useful temperature ranges (70–102 °C) has a significant drawback, this being low resistance to acids and water due to the imine chemistry. Moreover, in order to synthesize vanillin-based vitrimer via a catalyst-free imine pathway, extra chemical modification steps are required to convert the single aldehyde group in vanillin into multi-aldehyde-containing precursors. These modification steps are usually related to expensive and toxic chemicals. In contrast, in the transesterification approach, epoxidized vegetable oils can directly be used to produce vitrimers without the requirement for pretreatment. Nonetheless, this approach usually requires petroleum-based catalysts such as zinc acetate [Zn(OAc)_2_], zinc acetylacetonate anhydride [Zn(acac)_2_], tin(II) 2-ethylhexanoate [Sn(Oct)_2_], titanium(IV) isopropoxide [Ti(OPr)_4_], etc., which, in turn, reduce the sustainability of the process. Some other approaches such as disulfide, vinylogous urethane, transamidation, etc., require either catalyst or pretreatment steps or both, limiting their application to larger scales. As is evident from this review, transesterification and polyimine exchange are the two most common approaches to producing biobased and recyclable vitrimer materials which are closest to the market application, especially for epoxidized vegetable-oil- and vanillin-based vitrimers because both starting precursors are commercially available. However, the crucial challenges include pretreatment, processing, cost, properties, scalability, and environmental impact, leaving opportunities for future research and development. In conclusion, it is necessary for more research to be conducted to make bio-based vitrimers a viable next-generation solution for sustainable, recyclable, and high-performance materials.

## Data Availability

Data is contained within the article.

## Abbreviations

CFRC	Carbon fibre-reinforced composite
5-HMF	5-hydroxymethylfurfural
CA	Carboxylation
CANs	Covalent adaptable networks
CF	Carbon fibre
CMC	Carboxymethyl cellulose
CPA	Camphoric acid
DA	Dimer acid
DETA	Diethylenetriamine
DIFFA	Difurfurylamine
DSs	Degrees of substitution
EL	Enzymatic lignin
ELV	Enzymatic lignin-based vitrimer
ENR	Epoxy natural rubber
EP	Epoxidation
ESO	Epoxidized soybean oil
EVO	Epoxidized vegetable oil
FPA	Fumaropimaric acid
GAN	1,3-diaminoguanidine monohydrochloride
HCl	Hydrochloric acid
LEVs	Lignin-based epoxy vitrimers
NaOH	Sodium hydroxide
S/G	Syringyl/Guacyl
SAA	Starch acetoacetate
SA	Sebacic acid
TBD	1,5,7-triazabicyclo[4.4.0]dec-5-ene
T_g_	Glass transition temperature
TGDDM	N,N,N′,N′-tetraglycidyl-4,4′-diaminodiphenylmethane
THF	Tetrahydrofuran
VA	Vanillin
